# Heating Effect on the Liquid-Crystalline Octakis(hexylthio)zinc(II) Phthalocyanine Thin Film Sensor for the Detection of Chlorinated Hydrocarbon Vapors and Interaction Mechanism Analysis Using Density Functional Theory

**DOI:** 10.1021/acsomega.5c10679

**Published:** 2025-12-23

**Authors:** Zeynep Özer, Gizem Gümüşgöz Çelik, Inci Capan, Burcu Dedeoglu, Ayşe Gül GÜREK, Rifat Çapan

**Affiliations:** † 52962 Gebze Technical University, Science Faculty, Chemistry Department, 41400 Gebze, Kocaeli Turkey; ‡ 53003 Balikesir University, Science&literature Faculty, Physics Department, 10145 Balikesir, Turkey

## Abstract

A liquid-crystalline octakis­(hexylthio)­zinc­(II) phthalocyanine (**ZnPc2-B4**) spun thin film sensor was fabricated to study the heating effect on the detection of chlorinated hydrocarbon vapors and to identify their interaction patterns at the reactive sites between the **ZnPc2-B4** thin film and vapor molecules. The surface plasmon resonance (SPR) technique was employed to collect sensor response data, which were analyzed to determine the optical and sensor parameters. After the heating procedure, the thickness of the **ZnPc2-B4** thin film decreased from 49.5 to 43.5 nm, and the sensor sensitivity for dichloromethane vapor decreased from 0.2864 to 0.1015% response/ppm. A similar heating effect on the SPR curve measurement occurred, and the SPR curve was shifted from 0.12 ° to 0.09 ° when the **ZnPc2-B4** thin film was exposed to the dichloromethane vapor. Atomic force microscopy results showed a compact and uniform surface with a surface roughness value of 2.77 nm. Density functional theory was used to elucidate interaction patterns between the **ZnPc2-B4** thin film and the selected vapor. It was found that Zn···Cl, C···Cl, and N···H interactions occurred. In addition, the interaction of Zn with the nucleophilic carbon atom of the vapor interacts with the π-electrons of the C=C double bond. **ZnPc2-B4** spun thin film sensor could be a potential candidate for optical sensor applications, such as environmental monitoring or VOC detection.

## Graphical abstract

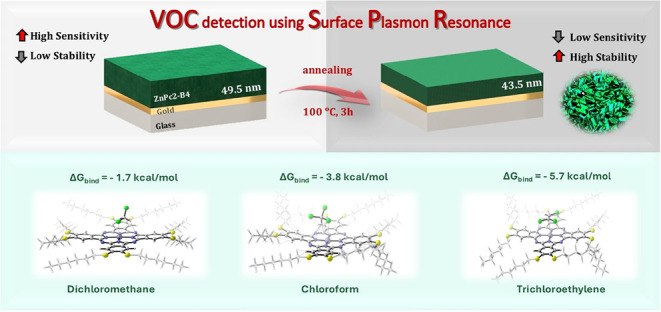

## 1 Introduction

Phthalocyanine (Pc) and metal phthalocyanine (MPc) derivatives in chemical sensing applications were widely used in a variety of industrial fields including liquid crystal applications, food, agriculture, security due to the insertion of metal ions or attachment of additional atoms or groups, their high thermal, chemical and physical stability, optical absorption and chemical functionality, environmentally friendly, and their unique electronic structure and properties.^1–3^ Pc material with a central metal atom plays a significant role in optical, electrical, and sensing properties due to the phthalocyanine crystal structure, and it provides an opportunity in the field of specific sensor applications.^4^ Several thin film methods, such as spin coating,^5^ Langmuir–Blodgett thin film technique,^6^ and organic molecular beam deposition,^7^ were used for Pc/MPc materials to fabricate a stable sensing element at room temperature in the field of gas sensor applications. Surface plasmon resonance (SPR) technique,^8^ is based on the excitation of surface electromagnetic waves of transverse magnetic modes traveling along the interface between a metal and a dielectric medium. It was introduced for optical characterization of organized thin films on a metal surface and was regarded as a powerful tool for monitoring changes in the thickness and the refractive index during the interaction between a thin film and gas molecules to be detected.

Vapor sensing properties of spin-coated thin films of mesogenic octasubstituted phthalocyanine derivatives were investigated using the SPR technique and Raman spectroscopy. The changes in the thickness, refractive indexes, and extinction coefficients of all MPc in response to chloroform, dichloromethane, benzene, and toluene vapors, and the thin film thickness were found to increase during vapor exposure, possibly due to the film swelling.^9^ In order to increase the sensor sensitivity for the detection of chloroform and benzene vapor, the copper phthalocyanine with eight decyltosylaminomethyl substituents was studied, and chloroform vapor yielded better sensor response than benzene.^10^ The interaction of chloroform vapor with MPc spun thin films was investigated by using Raman spectroscopy. It was found that there are no changes in the region of macrocyclic vibrations under chloroform exposure. However, the modes in the region of the C–H stretching vibrations of alkyl substituents become broader and shift to low-frequency regions. Hydrogen bonds occurred between hydrogen atoms of alkyl chains of the substituents and electron donor atoms (O, Cl) of these vapor molecules.^9^ The SPR response of the LB thin films of metal-free 2,3,9,10,16, 17,23,24-octakis­(octyloxy)-29*H*,31*H* phthalocyanine (H_2_Pc3c) and its zinc (ZnPc3c) and copper (CuPc3c) complexes mixed with stearic acid were tested to various VOCs (aromatic hydrocarbons, chlorinated hydrocarbons, and alcohols). This study concluded that the response to chlorinated hydrocarbons was noticeably higher than that of aromatic hydrocarbons and alcohols. The sensor response between LB films and organic vapors was explained as the formation of a hydrogen bond and/or a dipole/dipole interaction.^11^

The effect of the substituents in the ring of CuPc on the chloroform, dichloromethane, and toluene vapor sensing properties of their spun thin film sensors was investigated by the SPR technique. The SPR sensor response decreased with the expansion of the aromatic ring, and an increase in the alkyl-substituted length yielded an improvement in the SPR sensor response. The sensor interaction of CuPc spun films with organic vapors led to a change in both their thickness and optical parameters.^11^ ZnPc LB thin film was investigated for detecting tetrachloromethane, dichloromethane, m-xylene, and toluene vapors using SPR measurements. It was found that ZnPc LB thin film demonstrated a higher response to dichloromethane vapor than others.^12^ Other LB thin film vapor sensor studies were carried out by ZnPc bearing crown ether moieties against saturated acetone, methanol, ethanol, 2-propanol, and chloroform vapors. It was indicated that among the investigated vapors, the highest SPR sensor response was yielded for acetone, which has the largest dipole moment and diffusion coefficient.^6,13^

Several Pc, CuPc, and their derivatives were used to investigate the sensor properties of the VOCs. On the other hand, these thin films were directly linked to the crystal structure and existed in several molecular forms that range from amorphous to highly crystalline, depending on the deposition process, quality, orientation, and temperature of the solid substrate surface. The heating of the obtained MPc thin film, after the fabrication process, affected its crystalline form.^14^ This heating process was able to change the physical and chemical properties of MPc thin films used in the application of chemical sensors^15–17^ and vapor sensors.^18,19^

In recent years, liquid-crystalline metal phthalocyanine (LC MPc) materials as organic vapor sensors were studied. Some examples were listed as follows: LC NiPc thin film for chloroform and benzene vapor detection,^9^ hybrid LC ZnPc@Cu_2_O nanowires for ethanol vapor detection,^20^ LC CuPc and LC CoPc for ammonia,^18,21^ toluene, chloroform, CCl4, benzene, hexane, and methanol vapor detection.^17^

The spin-coating method provides a simple and convenient procedure for preparing ordered films of MPc materials, which can be heated to form thin LC films. There are limited studies on LC MPc spun films for an organic vapor sensor using the SPR technique. This article presents the results of SPR experiments for studying optical and sensor parameters of a spin-coated thin film of octakis­(hexylthio)­zinc­(II) phthalocyanine (**ZnPc2-B4**) material kept in an oven at 100 °C for 3 h. Using the SPR technique, the changes in optical and sensor parameters of LC **ZnPc2-B4** thin film sensors against dichloromethane, chloroform, and trichloroethylene vapors before and after the heating process are deeply investigated in terms of optical parameters (thickness, refractive index, and the extinction coefficient) and sensor parameters (response rate, sensitivity and limit of detection, quantification, reversibility and selectivity etc.). In addition, density functional theory (DFT) calculations are used to study the interaction mechanisms between the **ZnPc2-B4** thin film sensor and each vapor. Condensed Fukui functions are applied to identify their interaction patterns during the reactive sites between the **ZnPc2-B4** thin film and vapor molecules.

## 2 Experimental Details

### 2.1 Materials

4,5-Bis­(hexylthio)­phthalonitrile (PN2) and **ZnPc2-B4** material (Figure 1a) were synthesized and purified following previous work.^22^ The desired compound **ZnPc2-B4** was separated chromatographically from the reaction mixture. **ZnPc2-B4** showed high solubility in common organic solvents such as tetrahydrofuran, CHCl_3_, dimethylformamide (DMF), dimethyl sulfoxide (DMSO), CH_2_Cl_2_, and acetonitrile, thanks to the substitution with hexylsulfanyl units on the peripheral positions. This Pc derivative was fully characterized by using a variety of spectroscopic techniques, including NMR, FT-IR, mass spectrometry (MS), fluorescence, and electronic absorption spectroscopy; the analyses were consistent with the predicted structures. All other reagents were obtained from commercial suppliers.

**Figure 1:**
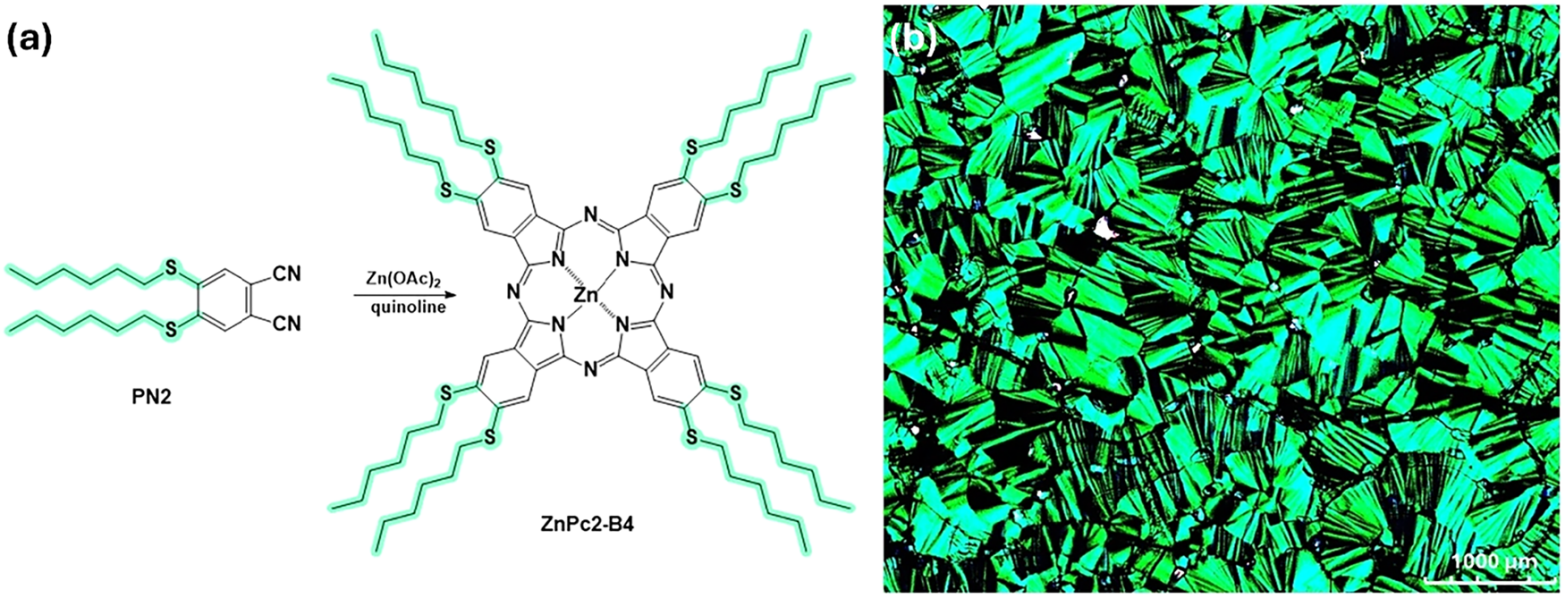
(a) Synthesis route of **ZnPc2-B4.** (b) Optical texture of **ZnPc2-B4** at 50 °C, in the Col_h_ mesophase (magnification 40×).

The mesogenic properties of the compound were previously described.^23^ Phase transition temperatures were established by differential scanning calorimetry (DSC) and polarizing microscopic observations for **ZnPc2-B4**. Optical texture was observed with the POM Biomed MMR-3. The compound **ZnPc2-B4** exhibits only one type of mesophase and forms the classical fan- or flower-like texture of the mesophases, indicating a columnar hexagonal mesophase (Col_h_) over a wide temperature range (7–300 °C)^23^ (Figure 1b).

### 2.2 The Preparation and Fabrication Process of the **ZnPc2-B4** Thin Film Sensor

The spin coating method was frequently used to fabricate organic thin films onto a gold-coated glass substrate, where it is suitable to use as a sensor element in the field of gas sensor applications.^24^ The fundamentals of the spin coating technique can be defined as applying a solution onto a solid substrate, which is capable of rotating about the horizontal axis at a desired rotation speed, resulting in the effect of the centrifugal force, and a uniform thin film on the solid substrate is formed. The precise control of its acceleration and deceleration can be achieved. A flexible and easily programmable equipment, Special Coating Systems (SCS) G3P-8 type spin coater system (USA), given in Figure 2, was used, which is adequate to deposit thin films with a superior level of reproducibility and thickness control onto a variety of plaque substrates; glass or gold-coated glass substrates. To prevent any dust that could contaminate the thin film structure, the spin coater had a glass-covered window to protect it. A disk-shaped vacuum holding platform was utilized to keep and support the substrate in place during the fabrication process, with up to 30 differing routes containing up to 20 steps. The SCS G3P-8 type spin coater system has no direct control of thickness; however, the solution concentration and the substrate rotation speed strongly affect the film thickness. This spin coater was employed to produce **ZnPc2-B4** thin film sensors against chloroform, dichloromethane, and trichloroethylene vapors.

**Figure 2:**
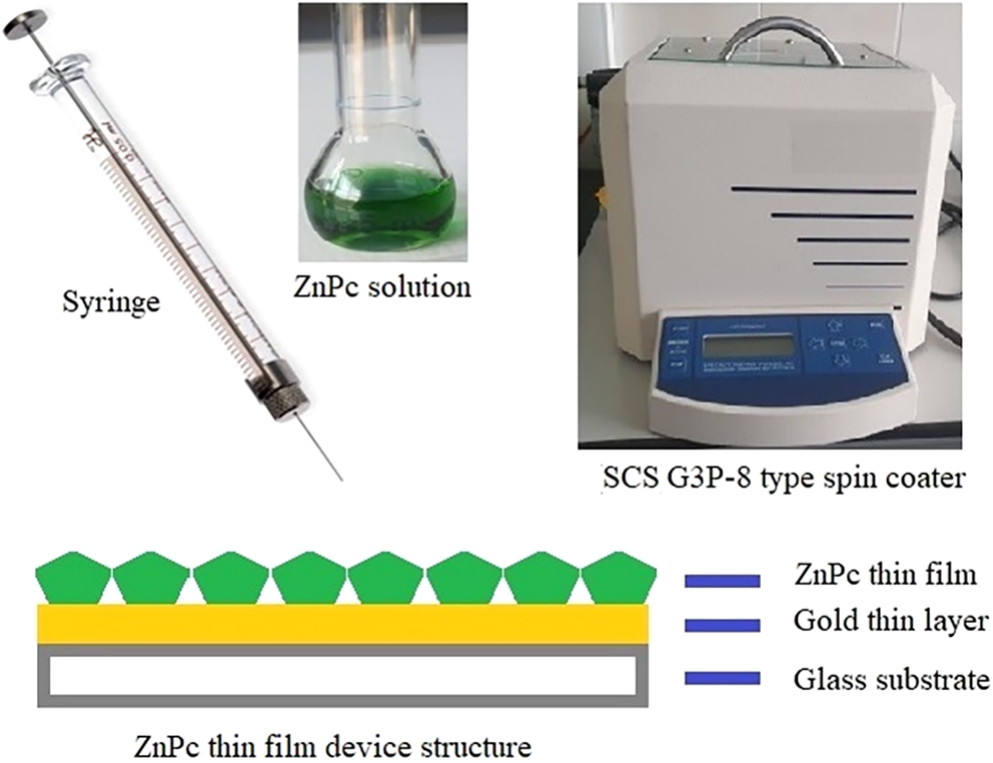
SCS G3P-8 spin coater system and fabrication process.

A concentration ratio of 1 mg/mL was obtained by dissolving the **ZnPc2-B4** compound using chloroform. A three-stage program was chosen for the film fabrication route. In the initial stage, the rotation speed of the rotating table with a solid substrate (20 mm × 20 mm × 1 mm glass slides coated with 50 nm gold, supplied by TEKNOTIP company in Turkey) in 10 s is reached to a value of 1000 rpm. At this constant spin speed, a volume of 100 μL of **ZnPc2-B4** solution was injected using a Hamilton microliter syringe onto the substrate, and the rotating table was kept rotating for another 35 s. In the final stage, the rotating table was decelerated to the initial inert situation during the next 10 s. The **ZnPc2-B4** thin film was then allowed to dry for 30 s. It is well-known that chloroform solutions evaporate quickly during film preparation; therefore, their effect on the thin film thickness is neglected. After the **ZnPc2-B4** films were produced, the MEMMERT heating oven was employed to heat from 25 to 100 °C. These films were kept at 100 °C for 3 h before they were slowly cooled back to 25 °C. The vapor sensing measurements for each thin film were recorded using the same laboratory conditions to compare the results before and after the heating process.

### 2.3 Vapor Sensor Measurements of the **ZnPc2-B4** Thin Film Sensor

Chloroform, dichloromethane, and trichloroethylene were purchased from Sigma-Aldrich (99%) and Thermo Scientific (99%), respectively, and used without further purification as the source of saturated organic vapors for **ZnPc2-B4** thin film sensor measurements.

The vapor concentrations (*c* in ppm) were calculated using eq 1 ^25^

1 $$c=\left(\frac{22.4\rho V}{{\mathrm{MV}}_{0}}\right)\times 10^{6}$$

where *c* (ppm) is the vapor concentration, *V* (5 mL) is the saturated vapor volume, ρ (g/mL) the saturated vapor density (1.489 g/mL for chloroform, 1.326 g/mL for dichloromethane, and 1.463 g/mL for trichloroethylene at 20 °C), *M* is the vapor molecular weight (119.37 g/mol for chloroform, 84.93 g/mol for dichloromethane, and 131.38 g/mol for trichloroethylene), and *V*_0_ (∼0.02 mL) is the volume of the gas cell.

To investigate the concentration dependence response of **ZnPc2-B4** thin films, four different concentration values (25, 50, 75, and 100%) of the saturated vapor/air ratios were chosen. In Table 1, the calculated concentration values in parts per million are presented. Concentration dependence measurements are crucial for determining sensor parameters, including sensitivity, the limit of detection (LOD), and the limit of quantification (LOQ). LOD is the lowest analyte concentration that can reliably be determined from the signal, where LOQ is the lowest concentration of a substance that can be measured with certainty using the standard test.

**Table 1:** Concentration Values in ppm

	25% (1.25 mL)	50% (2.5 mL)	75% (3.75 mL)	100% (5 mL)
VOCs	concentration (ppm × 10^3^)			
Chloroform	17.47	34.94	52.40	69.87
Dichloromethane	21.87	43.74	65.60	87.47
Trichloroethylene	15.59	31.18	46.77	62.36

A BIOSUPLAR 6 Model spectrometer, given in Figure 3a, was employed to collect vapor sensor data when the **ZnPc2-B4** thin film interacted with selected vapor molecules. It was utilized by a low-power (630–670 nm) laser diode as the light source, with an angular resolution of approximately 0.003 °. Biosuplar-software was used to collect data and to control the instructions to operate the SPR instrument. The reflected light intensity versus incidence angle (SPR curves) and the intensity versus time at a fixed angle (kinetic measurement) were recorded for gas sensing investigations. The 50 nm thick gold-coated glass slides were set onto a glass prism (*n* = 1.51), where the index-matching fluid ethyl salicylate was used to provide the optical contact between the slide and the prism. Afterward, the prism was mounted onto the holder of the SPR system. A total internal reflection was obtained by the interaction of the polarized beam of laser with the gold layer, the interface between the gold layer, and its coating of **ZnPc2-B4** thin films. The reflected light intensity is not dependent on the incidence angle of total internal reflection. However, a surface plasmon resonance is formed when the incidence is greater than the critical angle, accompanied by the excitation of the delocalized electrons at the surface of the gold layer. The SPR resonance angle is defined at this critical angle, leading to a sharp reduction in the reflected light intensity because of the adsorption of the energy.

**Figure 3:**
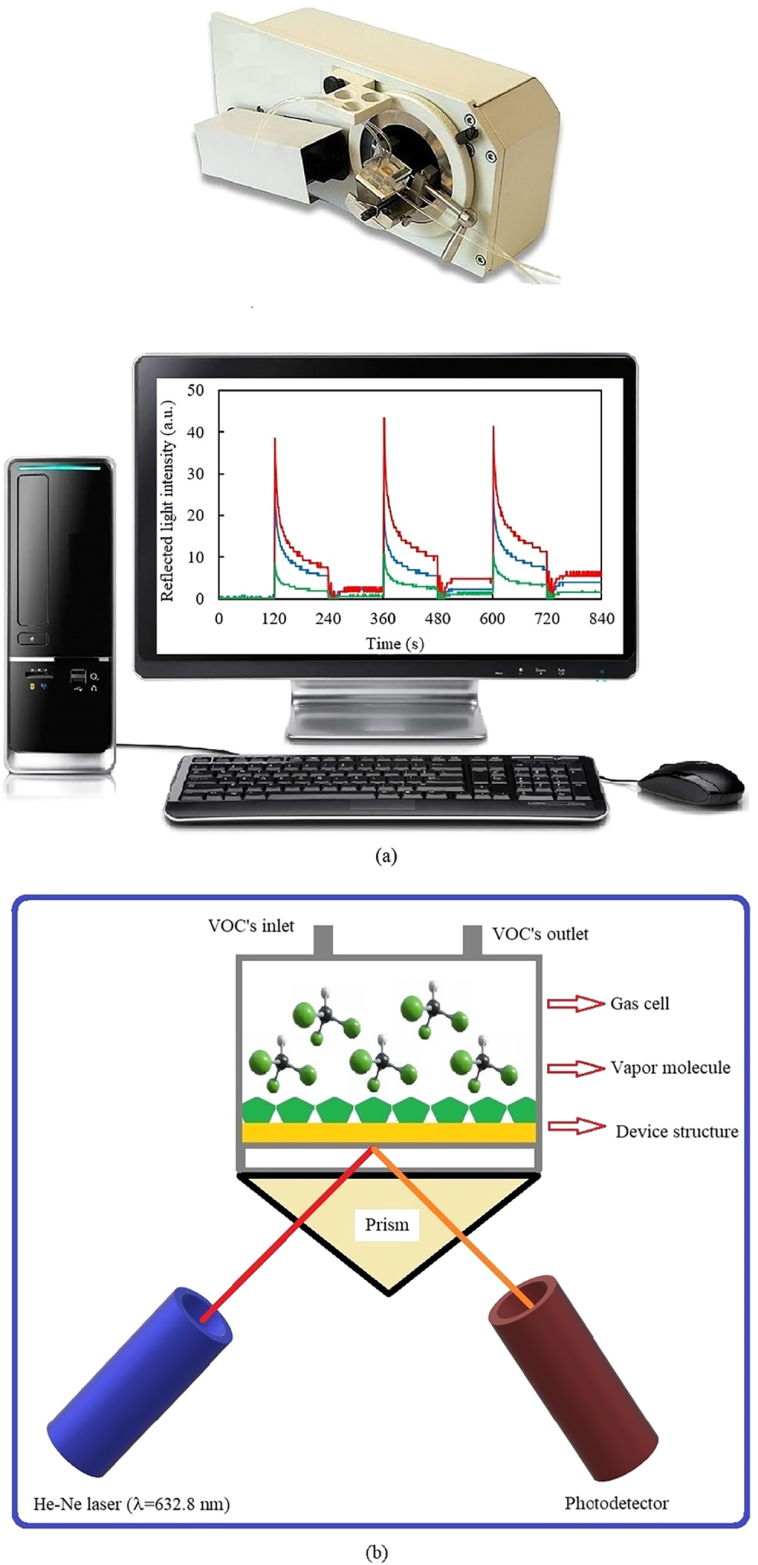
(a) View of the BIOSUPLAR 6 Model SPR spectrometer. (b) Schematic diagram of the SPR gas cell.

A diagram of our SPR gas cell is presented in Figure 3b. The gas cell, consisting of an outlet and an inlet of analyte gas, which are both fixed with and connected to silicone tubes, was made of transparent plastic. **ZnPc2-B4** thin films on the gold-coated glass slides were placed between the gas cell and the prism for the gas sensing experiments, which were performed by injection of the vapor into the gas cell using a syringe. The light intensity at the fixed angle (θ) was measured in real time while the interactions between the thin layer and the organic vapors (chloroform, dichloromethane, and trichloroethylene) occurred.

Concentration dependence behavior is important in sensor applications to determine the sensitivity of an applied sensor. In this research, an investigation of the concentration dependence of sensor behavior between the **ZnPc2-B4** thin films and chlorinated hydrocarbon vapor was carried out at room temperature (25 °C).

Each chlorinated hydrocarbon in the liquid phase was half-filled into glass tubes, which were heated with a hot water pocket to assist the stimulation to reach equilibrium and obtain saturated vapor. A 5 mL syringe was used to inject the saturated analyte with concentration vapor/air ratios of 25, 50, 75, and 100% into the gas cell. The tested vapors were injected into the gas cell, allowing them to interact for 2 min with the **ZnPc2-B4** thin layer. Another period of 2 min was followed by injecting dry air afterward. An ideal baseline signal should be obtained using high-vacuum media. In order to obtain a baseline, the gas cell was purged with dry air for two minutes. Three repeated successive kinetic measurements were recorded at a (100%) concentration value for each saturated vapor to test the stability and reproducibility of the **ZnPc2-B4** thin films, all kinetic measurements were performed at 25 °C temperature and at 25% Relative Humidity (RH) as follows using an HTC-2 LCD Digital Thermometer. In this study, the film thicknesses are estimated by the recorded experimental SPR curve data via fitting using WINSPALL fitting software (developed by Wolfgang Knoll, at the Max-Planck Institute for Polymer Research, Germany).^26,27^

## 3 Results and Discussion

### 3.1 Surface Morphology Analysis by Atomic Force Microscopy

The surface morphology of the **ZnPc2-B4** thin film after thermal annealing was examined by using atomic force microscopy (AFM) to assess nanoscale structural features. Figure 4 displays the AFM topography image obtained from a 10 μm × 10 μm scan area. The film shows a compact and uniform surface with fine, grain-like features distributed across the scanned region. Surface roughness measurements, which are characterized by the root-mean-square roughness (RMS) (Rq) value of 2.77 nm, an average roughness (Ra) of 1.52 nm, and a peak-to-valley distance (Rz) of 12.90 nm, corresponding to moderate topographical variation. The overall vertical profile spanned approximately 14.7 nm. This level of roughness suggests that the film surface remains smooth enough to ensure structural integrity while providing a sufficient surface area for vapor interaction.

**Figure 4:**
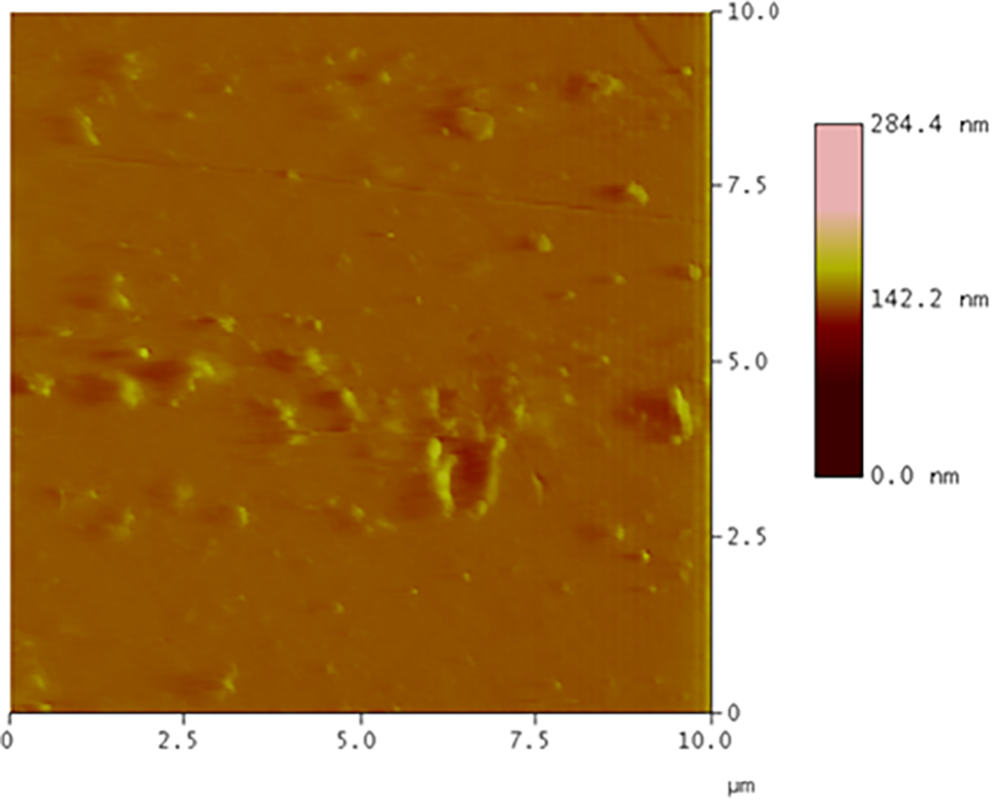
AFM topography image of the **ZnPc2-B4** thin film after annealing.

### 3.2 SPR Curve Measurements of the **ZnPc2-B4** Thin Film Sensor

In this section, SPR curves are used to determine the optical constants of the **ZnPc2-B4** thin film before and after heating. The reflected light intensity data were collected as a function of the angle of incidence with an accuracy of 0.003 °. SPR curve results are listed in Figure 5. The WINSPALL fitting program is applied to these experimental data to determine the **ZnPc2-B4** film thickness (*d*), refractive index (*n*), and extinction coefficient (*k*). Fitting results are listed in Table 2.

**Figure 5:**
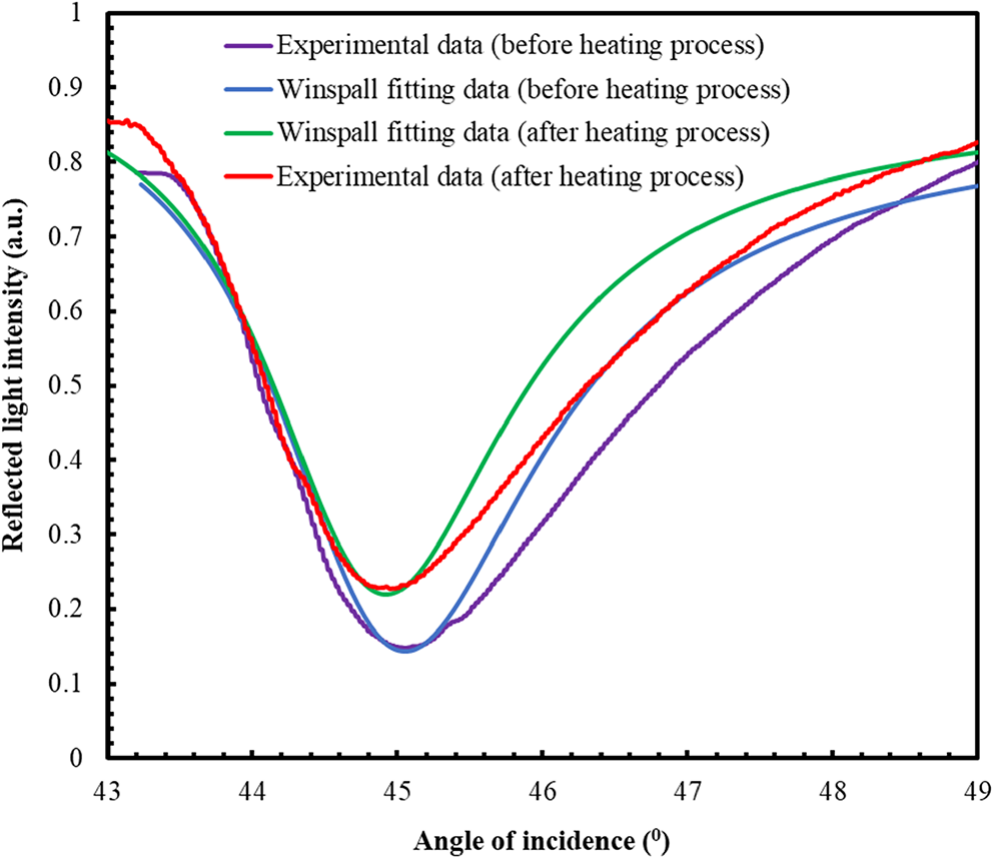
SPR experimental and fitting curves of the **ZnPc2-B4** thin film.

**Table 2:** Calculated Optical Constants of the **ZnPc2-B4** Thin Film

The **ZnPc2-B4** thin film sensor	*d* (nm)	*n*	*k*
before heating	49.5	1.52	0.1154
after heating	43.5	1.54	0.1286

The refractive index of the deposited thin film is found to be compatible with the literature^28^ under investigation using the laser beam with a wavelength of 632.8 nm. Moreover, similar studies have shown the refractive index values ranging between 1.41 and 1.72 of the Pc thin films fabricated using several thin film fabrication techniques, such as LB^12^ and physical vapor deposition.^29^ The thickness and optical constants of the thin films were also investigated via WINSPALL software, and the results showed thinner thin films after the heating procedure. A decrease in the thickness of the thin film, which is believed to be a result of the heating procedure, was observed. An increase in both refractive index and the extinction coefficient after the heating procedure was accompanied by this, as given in Table 2.

The results in the literature for as-deposited and heated thin films fabricated via spin coating and LB thin film fabrication techniques show similar behavior,^30^ which is believed to be a result of the aggregation of the molecules with the effect of heat. Changes in the optical constants (*n* and *k*) were also observed for the thermally evaporated **ZnPc2-B4** thin films upon annealing via structural transformation. Popielarski et al. investigated the influence of heat treatment on the structural and optical properties of NiPc and CuPc thin films in the field of OLED technology. Their Raman spectra proved that the physical properties of the studied NiPc and CuPc thin layers are closely related to heat treatment, and this heat treatment increased the refractive index and the extinction coefficient values of these thin films.^14^

Figure 6 shows a typical SPR curve obtained for the **ZnPc2-B4** thin film sensor before and after exposure to dichloromethane vapor. It is debatable that an angle shift in the SPR minimum occurred due to the adsorption of dichloromethane vapor on the **ZnPc2-B4** thin film sensor before and after the heating process. On injection of dry air into the gas cell, the recovery of the **ZnPc2-B4** thin film sensor to dichloromethane is found to be almost reversible.

**Figure 6:**
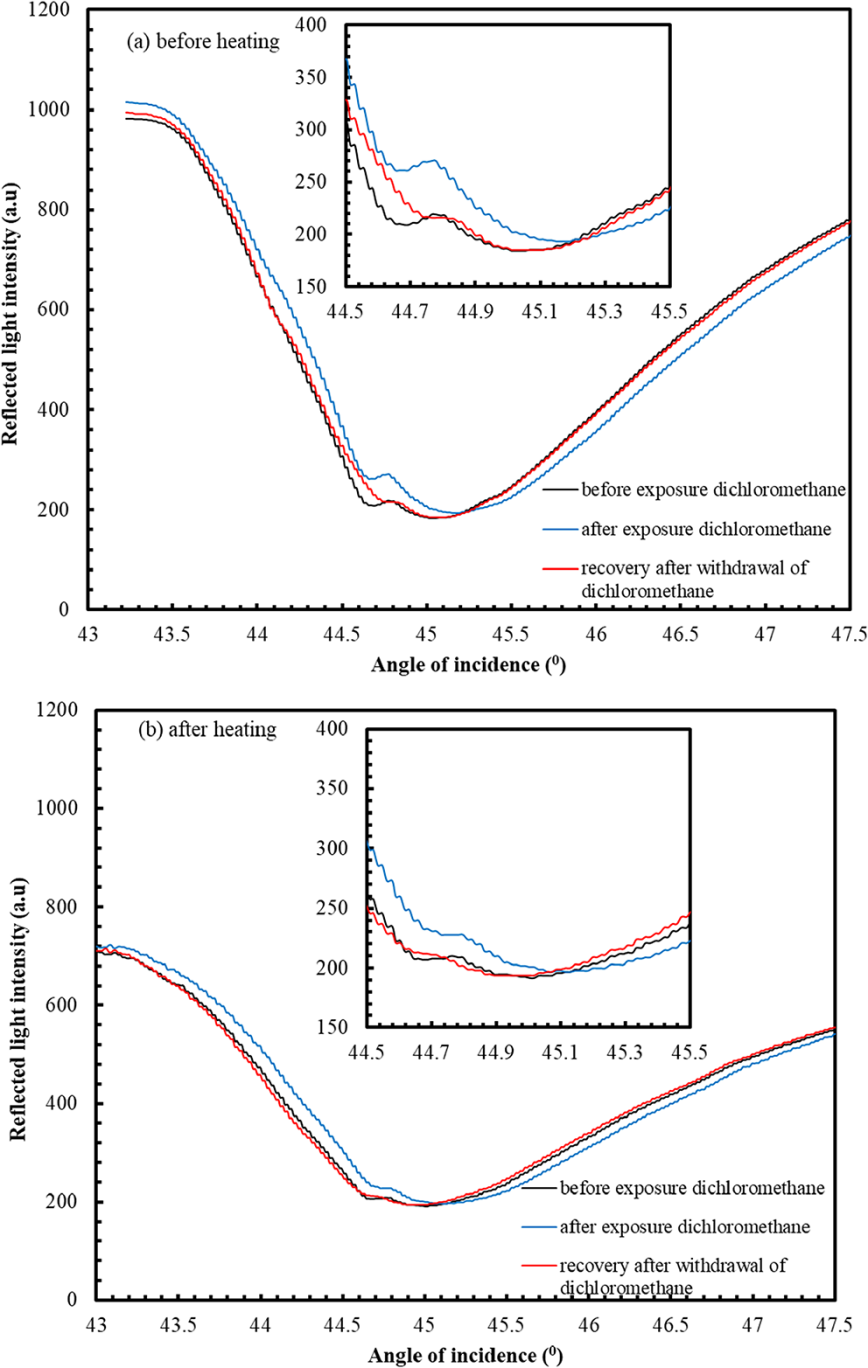
SPR curves obtained for the **ZnPc2-B4** thin film sensor, (a) before and (b) after the exposure to dichloromethane vapor.

The same measurements were carried out for other chloroform and trichloroethylene vapors at the same vapor/air ratio concentrations. The Δθ values of the **ZnPc2-B4** thin film before and after the heating process are presented in Figure 7.

**Figure 7:**
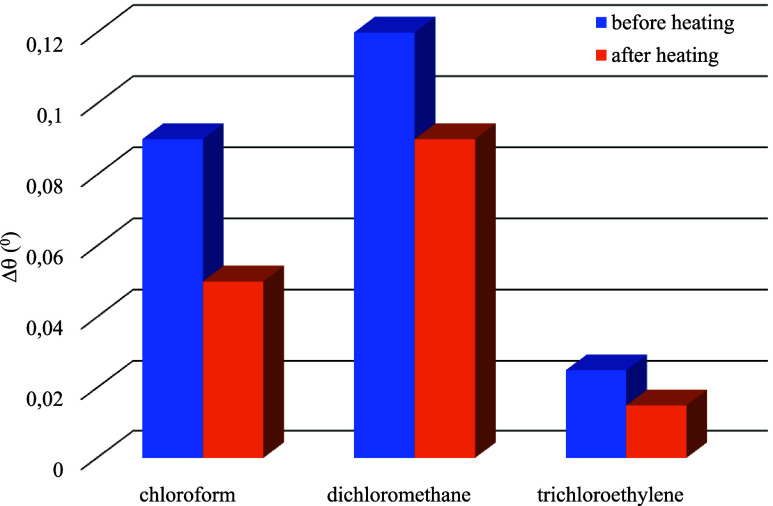
Δθ values of the **ZnPc2-B4** thin film sensor on exposure to different vapors.

According to the Δ*q* values, the **ZnPc2-B4** thin film sensor is reasonably selective for dichloromethane vapor over the others. The Δ*q* values were decreased after the heating process of the **ZnPc2-B4** thin film sensor due to a change of thickness value from 49.5 to 43.5 nm and a change of refractive index value from 1.52 to 1.54. The right shift of the SPR minimum is explained in terms of an increase in the change in the film thickness and the refractive index of the sensitive layer due to the film swelling.^31^ The refractive index of chloroform is 1.4459, a value close to that of the **ZnPc2-B4** thin film sensor, and the effect of chloroform adsorption on the refractive index is not expected to be significant. Similar refractive index values are 1.4242 for dichloromethane and 1.4777 for trichloroethylene, respectively. It is therefore believed that the resonance angle shift is primarily caused by the change in the film thickness due to the swelling process. Similar results have been found with ellipsometry fitting procedures using a copper octakisalkylthiophthalocyanine (C6S)­8PcCu spun thin film deposited at 2000 rpm. The thickness value was decreased from 56.3 to 52.2 nm, the refractive index value and the extinction coefficient were increased from 1.35 to 1.39 and from 0.25 to 0.30, respectively.^32^

### 3.3 Real-Time SPR Kinetic Measurements of the **ZnPc2-B4** Thin Film Sensor

In a sensor investigation, several parameters such as response rate, concentration dependence, response and recovery time, selectivity, sensitivity, stability, LOD, and LOQ should be investigated. Here, these sensor parameters for the **ZnPc2-B4** thin film sensor before and after heating against selected vapors are determined using real-time SPR kinetic measurements. To study concentration dependence behavior, a 5 mL syringe was used to inject each vapor into the gas cell with the concentration vapor/air ratio of 25, 50, 75, and 100% within a 2 min time interval. Real-time kinetic responses of the **ZnPc2-B4** thin film sensor to the selected vapor were recorded by measuring the reflected light intensity at a constant angle. Figure 8 shows the concentration dependence response of the reflected light intensity as a function of time.

**Figure 8:**
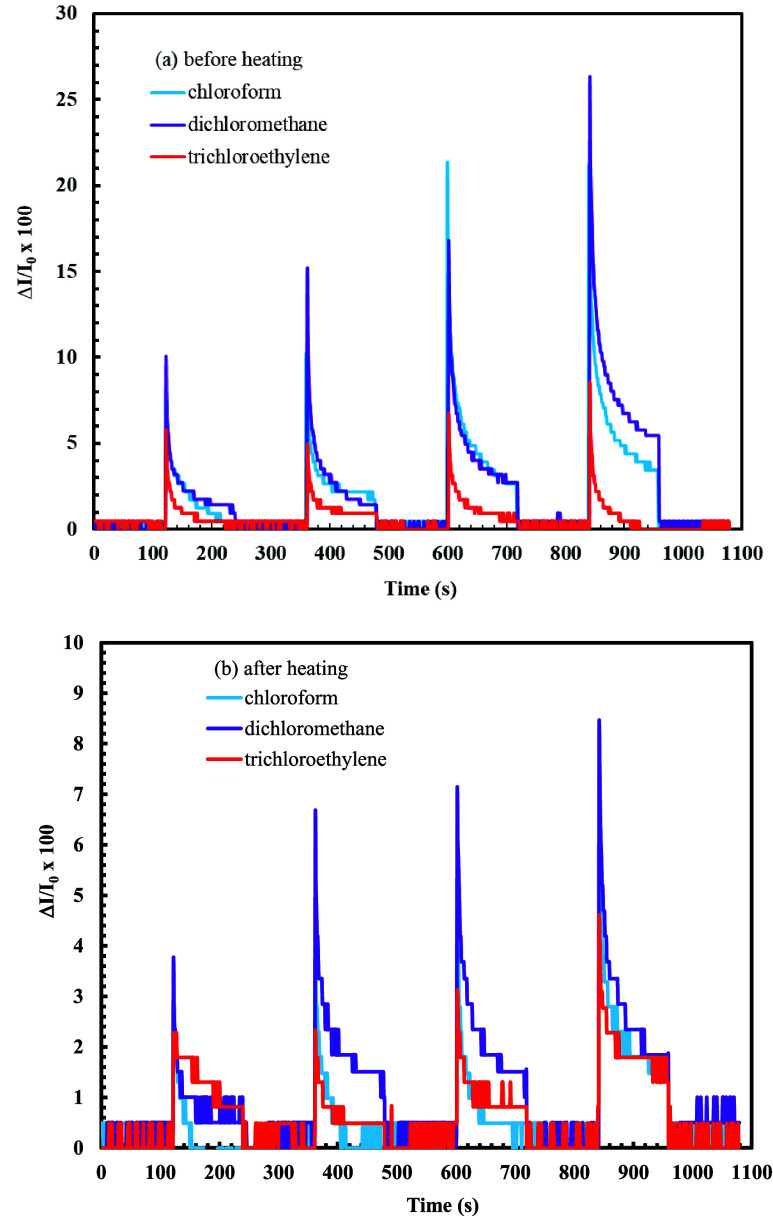
Concentration dependence behavior of the **ZnPc2-B4** thin film sensor a) before heating b) after heating.

The difference in the real-time kinetic measurement data before the exposure of a thin film sensor to selected vapor and during exposure to the same vapor is expressed as

2 $$\Delta I=\left(I-I_{o}\right)$$

where the reflected light intensities at the stationary and the vapor interaction stage are *I* and *I*_o_, respectively. Sensor response rate against selected vapor in a real-time kinetic study is described as^24^

3 $$\mathrm{sensor}\;\mathrm{response}\left(\%\right)=\frac{\Delta I}{I_{o}}\times 100$$

Response and recovery times are important sensor parameters because they represent the working speed of a sensor. The response time, which is well-known as the time required for the beginning of the interaction process, was characterized by the absence of a change in reflected light intensity. Recovery time was described as the time required to return to the initial reflected light intensity value known as the baseline. Both the response time and the recovery time of a sensing element indicate the speed of operation of the vapor sensor from the time when the target gas is exposed or removed.

Reproducibility, stability, and sensitivity behavior of the **ZnPc2-B4** thin film sensor before and during the heating process describe the consistency of vapor sensing response after continuous vapor sensor testing at the same concentration. If a sensor yields the same response for each cycle as a function of time, this sensor is called reproducible and stable. The selectivity of a sensor to a specific vapor must be detailed in sensor research. In a vapor mixture, how a sensor responds to a particular vapor can be decided by selectivity. It can be described as the proficiency of vapor sensors to detect specific vapors.

Figure 9 shows the reproducibility, stability, and sensitivity measurements of the **ZnPc2-B4** thin film sensor. For the reproducibility and stability test of the **ZnPc2-B4** thin film sensor, three reciprocal cycles of kinetic measurements were recorded at a constant (100%) concentration value for each saturated vapor.

**Figure 9:**
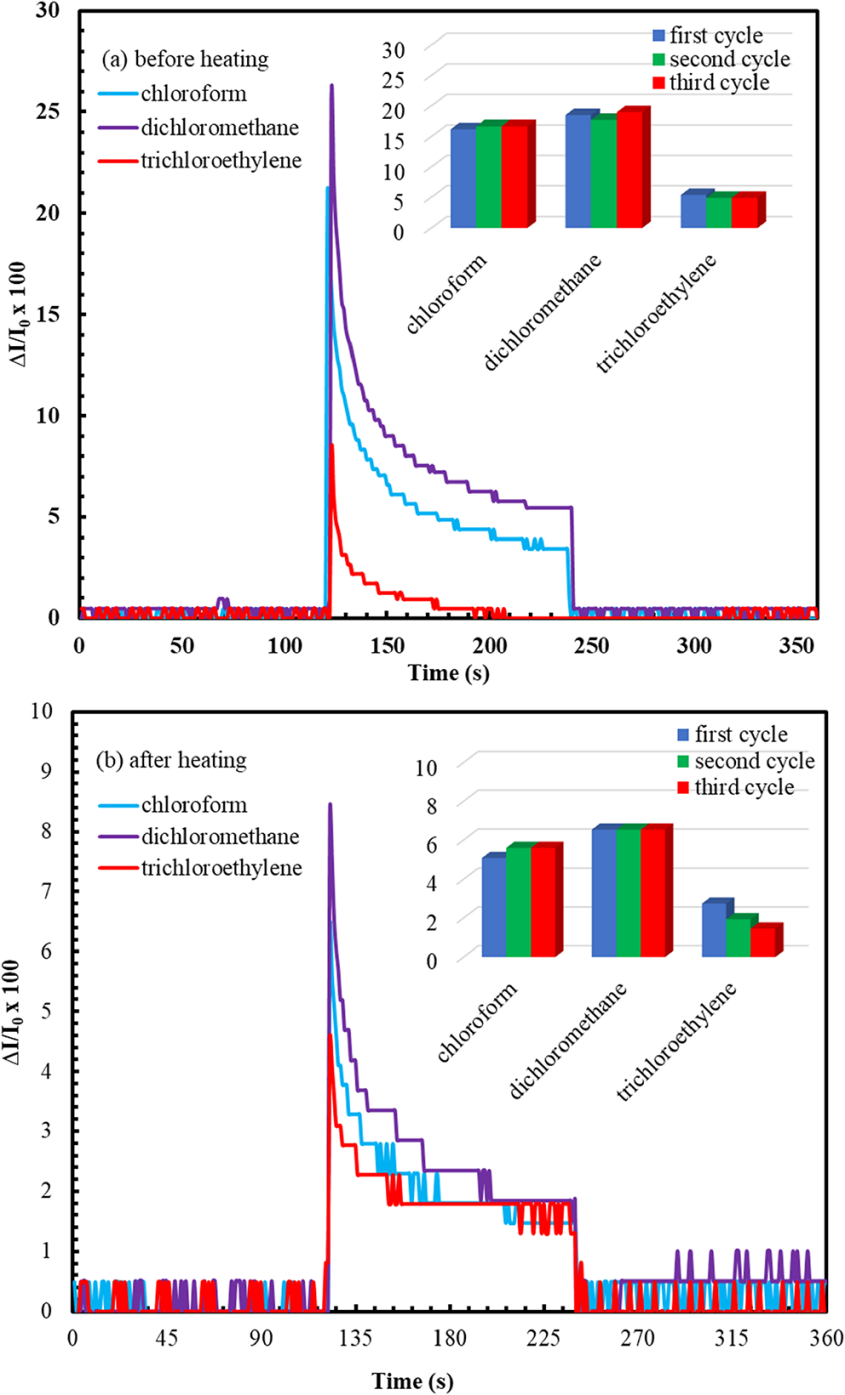
Reproducibility, stability, and sensitivity measurements of the ZnPc2-B4 thin film sensor a) before heating b) after heating..

Before and after the heating process, the **ZnPc2-B4** thin film sensor was tested against dichloromethane, chloroform, and trichloroethylene vapors. As can be seen from Figure 9, the response order was obtained as dichloromethane, chloroform, and trichloroethylene. A similar situation was observed in the measurements made after the heating process. According to the results obtained, it was concluded that the **ZnPc2-B4** thin film sensor was more selective against dichloromethane vapor than the others. Similar results were found after the heating process; however, the heating process reduced the response of the **ZnPc2-B4** thin film sensor. In the thickness measurements using WINSPALL software and SPR experimental data, it was revealed in the calculations that there was a decrease in the thickness of the **ZnPc2-B4** thin film sensor after the heating process. It is well-known that the interaction between the sensor element and vapor molecules in the SPR system is highly dependent on the thickness change. Therefore, it was evaluated that the decrease in the sensor response after the heating process was due to the thickness change of the **ZnPc2-B4** thin film sensor. The thickness dependence of the **ZnPc2-B4** thin film sensor response was available in the literature.^10^ It is found that the sensor response was due to a change in the optical characteristics of **ZnPc2-B4** thin film during adsorption of molecules of vapor under study as a result of van der Waals contacts with atoms of substituents.^1^

For the reproducibility and stability tests, time-dependent SPR kinetic measurements of the **ZnPc2-B4** thin film sensor were taken for 3 cycles at a constant concentration before and after heating. The inset graphs in Figure 9 show the sensor response to the vapors. Response values calculated using eq 3 were similar for the reciprocal exposures of the saturated concentration of each vapor. The reproducible response was obvious, where the recovery of the sensor was also tested. Full recovery of the sensor was observed. The reproducible character of the sensor with full recovery was relevant for both the as-prepared and heated sensor.

Sensitivity value (*S*) is one of the most important parameters used to analyze the sensor performance. By plotting the response of the sensor versus the concentration value of the target VOC molecule, we can use the slope of the linear graph to calculate the *S* value. It can be formalized as

4 $$S=\frac{\mathrm{sensor}\;\mathrm{response}}{\Delta C}$$

where Δ*C* is the change in the concentrations of organic vapor.

Low limit of detection (LOD) and limit of quantification (LOQ) values are other important sensor parameters that indicate the lowest amount of the analyzed vapor that can be detected by a sensor and the lowest amount of the analyzed vapor that can be accurately quantified, respectively.^33^ They proved the sensitivity and precision of a sensor element. The LOD value is described as^34^

5 $$\mathrm{LOD}=\frac{3.3\sigma }{S}$$

where σ is the standard deviation for our SPR measurements (0.001), and *S* is the sensitivity. LOQ value is described for the **ZnPc2-B4** thin film sensor as^35^

6 $$\mathrm{LOQ}=\frac{10\sigma }{S}$$

Figure 10 shows the change in the **ZnPc2-B4** thin film sensor response depending on the concentration before and after the heating process. The results obtained showed that the **ZnPc2-B4** thin film sensor response changed depending on the concentration and had different slopes for the three selected vapors. The slopes of these graphs are used to determine the *S* and fitting correlation (*R* ^2^) values. S, LOD, LOQ, and *R* ^2^ values were calculated by using the data given in Figure 8 and eqs 4–6. All obtained values are summarized in Table 3.

**Figure 10:**
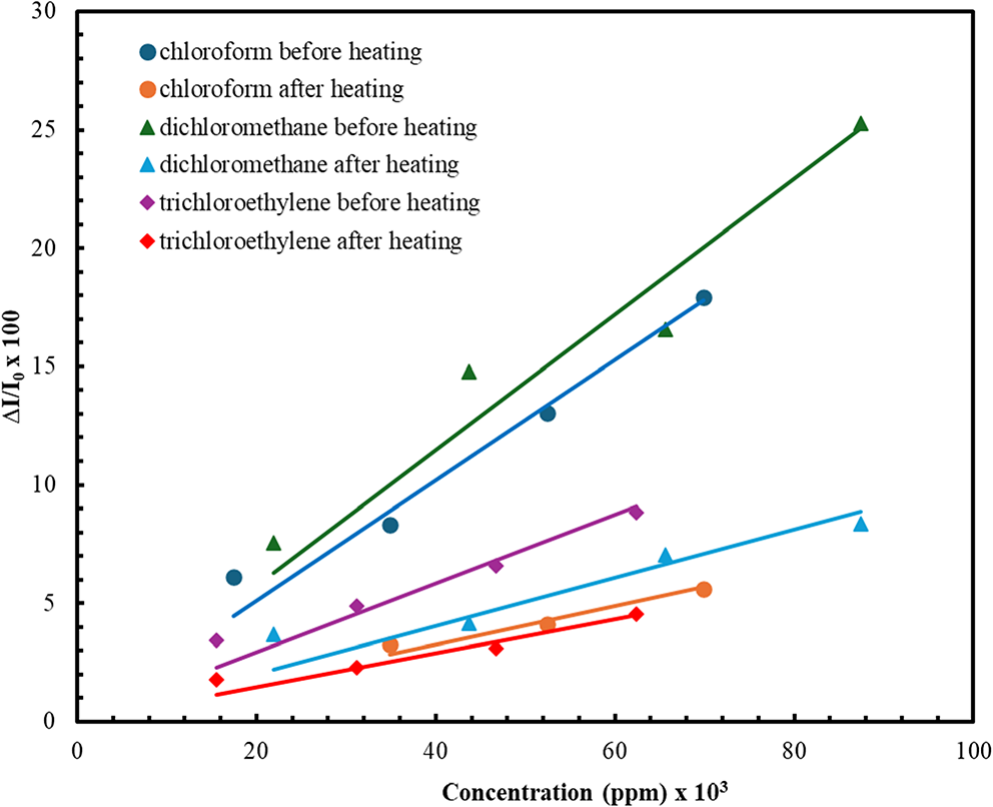
Concentration-dependent sensor response of the **ZnPc2-B4** thin film.

**Table 3:** Sensor Parameters of the **ZnPc2-B4** Thin Film

	sensitivity × 10^‑3^ (%response/ppm)		response/recovery times (s)		LOD (ppm)		LOQ (ppm)		*R* ^2^	
sensor parameters	before heating	after heating	before heating	after heating	before heating	after heating	before heating	after heating	before heating	after heating
Chloroform	0.2550	0.0814	1/9	1/8	12.94	40.54	39.21	122.85	0.9946	0.9962
Dichloromethane	0.2864	0.1015	2/7	1/7	11.53	32.51	34.96	98.52	0.9900	0.9827
Trichloroethylene	0.1461	0.0724	1/10	2/10	22.58	45.58	68.44	138.12	0.9898	0.9864

The *S* values decreased after the heating process for all vapors. Dichloromethane vapor showed a higher response than the others before and after the heating process. As a result, the **ZnPc2-B4** thin film sensor response decreased for all three vapors after the heating process. Similar changes were observed in the LOD and LOQ values. The increase in the LOD and LOQ values after the heating process was interpreted as decreasing the **ZnPc2-B4** thin film sensor performance. The reason for the decrease in the **ZnPc2-B4** thin film sensor performance after the heating process could be related to the decrease in film thickness observed in the SPR measurements. It can be concluded that the decrease in the film thickness affected the sensor performance. A similar study was also observed in the literature.^36^ It is well-known that heat treatment causes the structural transformation of Pc thin films. The annealing effects of CuPc thin film on the film characteristics and sensing properties were studied, and it is observed that the film sensitivity and response rate of the sensing experiment are decreased after heat treatment due to the reduction in the surface area. The CuPc thin films without heat treatment have a short response time and higher sensitivity due to the higher surface area of the fine-grain structure of the deposited films.^16,37^ Both studies have good agreement with our results, and they indicate that after heat treatment, the grains grow larger, and a more compact structure is formed. These researchers concluded that although heat treatment always leads to a smaller sensitivity, especially for the CuPc sensor, appropriate heat treatment requires consideration of the gas recovery process and the film stability.

### 3.4 DFT Analysis and Theoretical Calculation on Film-Vapor Interactions

To understand the nature of the binding capability of the **ZnPc2-B4** thin film sensor with chloroform, dichloromethane, and trichloroethylene vapors, geometric optimizations and theoretical calculations for interaction mechanisms were verified by applying DFT analysis. All geometries were optimized at the B3LYP/def2-SVP level of theory with Grimme’s D3 dispersion correction, as implemented in Gaussian 16.^38^ Condensed Fukui functions were used to identify the reactive sites in **ZnPc2-B4** and VOCs, and to predict their interaction patterns during complex formation. Table 4 presents the atoms in **ZnPc2-B4** and VOCs with the highest Fukui indices. The *f*+ values indicate electrophilicity and correspond to sites prone to nucleophilic attack, while the f values represent nucleophilicity and correspond to sites susceptible to electrophilic attack. Zn in **ZnPc2-B4** was found to be the most electrophilic site (*f*+ = 0.63), while the nitrogen atoms of the phthalocyanine displayed significant nucleophilicity (*f*– = 0.34). In the VOCs, chlorine atoms were typically the most nucleophilic, with dichloromethane showing the highest *f*– value (0.52), and hydrogen atoms commonly exhibited a mild electrophilic character.

**Table 4:** Condensed Fukui Function Values (*f*+ and *f*−) for the Most Reactive Atoms in **ZnPc2-B4** and VOC Molecules

compound	atom	*f*+	atom	*f*-
**ZnPc2-B4**	Zn	0.63	N	0.34
Dichloromethane	H	0.15	Cl	0.52
Trichloroethylene	H	0.16	C	0.35
Chloroform	H	0.16	Cl	0.34

The formation of **ZnPc2-B4** -VOC complexes is governed by complementary interactions between these nucleophilic and electrophilic centers. For each **ZnPc2-B4**–VOC, the most stable complex structure is illustrated in Figure 11. The binding energies of the resulting complexes are reported in kcal/mol along with the corresponding interaction distances in angstroms (Å).

**Figure 11:**
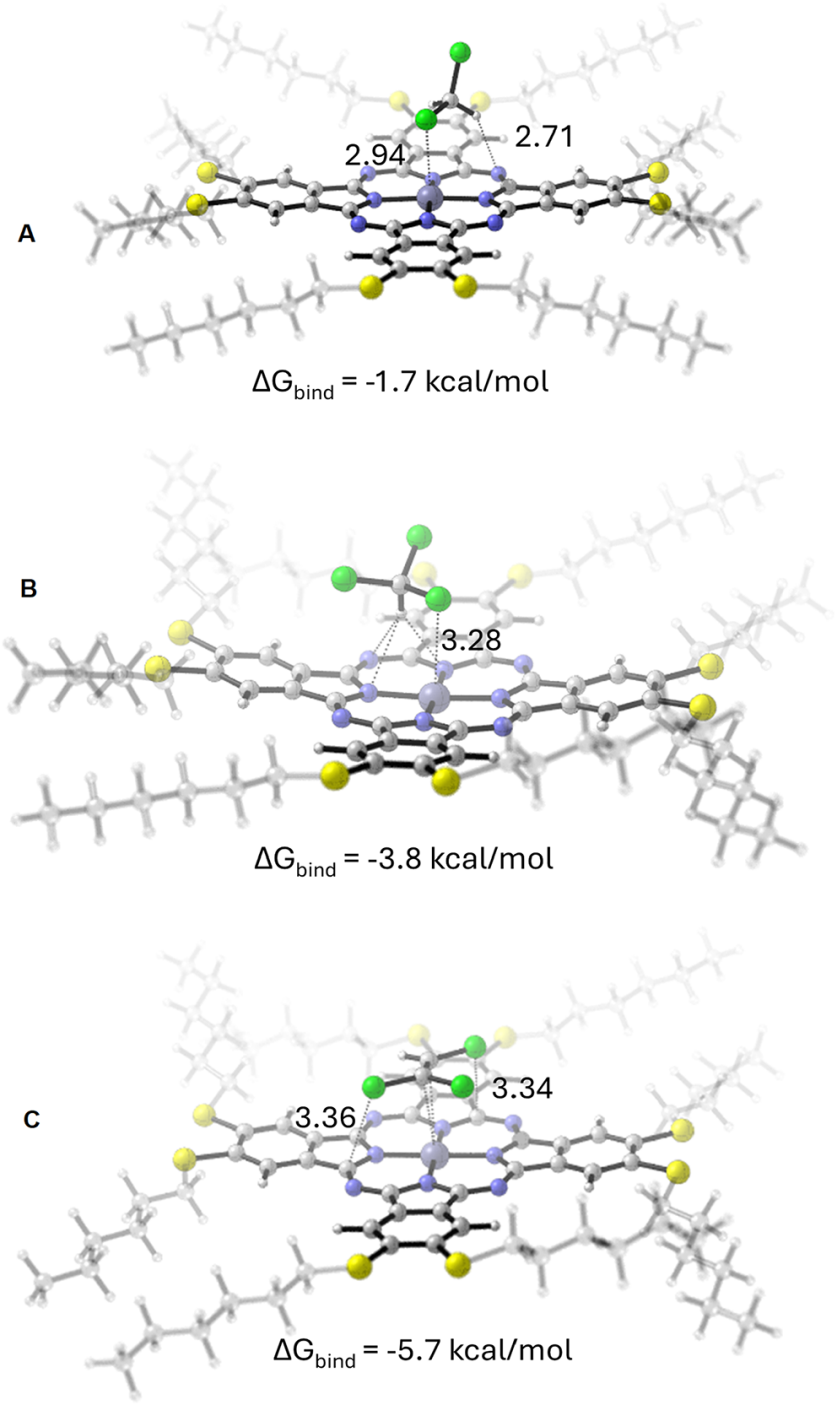
Optimized geometries of the most stable **ZnPc2-B4**–VOC complexes: (A) dichloromethane, (B) chloroform, and (C) trichloroethylene. Key intermolecular interactions are shown with dashed lines along with their corresponding distances in angstroms (Å).

In the **ZnPc2-B4–**dichloromethane system, the most stable complex is characterized by an interaction between the electrophilic Zn center of **ZnPc2-B4** and the nucleophilic chlorine atom of dichloromethane (*f*– = 0.52). The optimized structure exhibits Zn···Cl distances of 2.94 Å with a binding free energy of −1.7 kcal/mol. Additionally, the complex is stabilized by secondary N···H interactions.

For the **ZnPc2-B4–**chloroform complex, stabilization arises from the Zn···Cl interaction, where the chlorine atom of chloroform possesses a nucleophilic Fukui index of 0.34. The Zn···Cl distance in the optimized structure is 3.28 Å, and the calculated binding energy is −3.8 kcal/mol. Similar to chloroform, this complex is stabilized by secondary N···H interactions.

In the case of **ZnPc2-B4–**trichloroethylene, the most stable configuration involves the interaction of Zn with the nucleophilic carbon atom of trichloroethylene (*f*– = 0.35). Here, Zn interacts with the π-electrons of the C=C double bond. The complex is further stabilized by C···Cl interactions with distances of 3.36 and 3.34 Å and a binding energy of −5.7 kcal/mol. The strong binding is attributed to the effective frontier orbital overlap and favorable electrostatic complementarity between the reactive sites.

The HOMO–LUMO analysis was conducted to understand the electronic features of **ZnPc2-B4**–VOC complexes (Figure 12). In all cases, HOMO is mainly localized on the **ZnPc2-B4** macrocycle, indicating its electron-donating character. The LUMO is typically centered on the Zn atom and extends toward the interacting VOC molecule, supporting **ZnPc2-B4**’s role as an electrophilic acceptor. Among the systems, the **ZnPc2-B4**–trichloroethylene complex shows the most significant HOMO–LUMO interaction, consistent with its stronger binding energy. The orbital distribution in this complex suggests better charge transfer and electronic complementarity compared to those of chloroform and dichloromethane complexes, which show weaker and more localized interactions.

**Figure 12:**
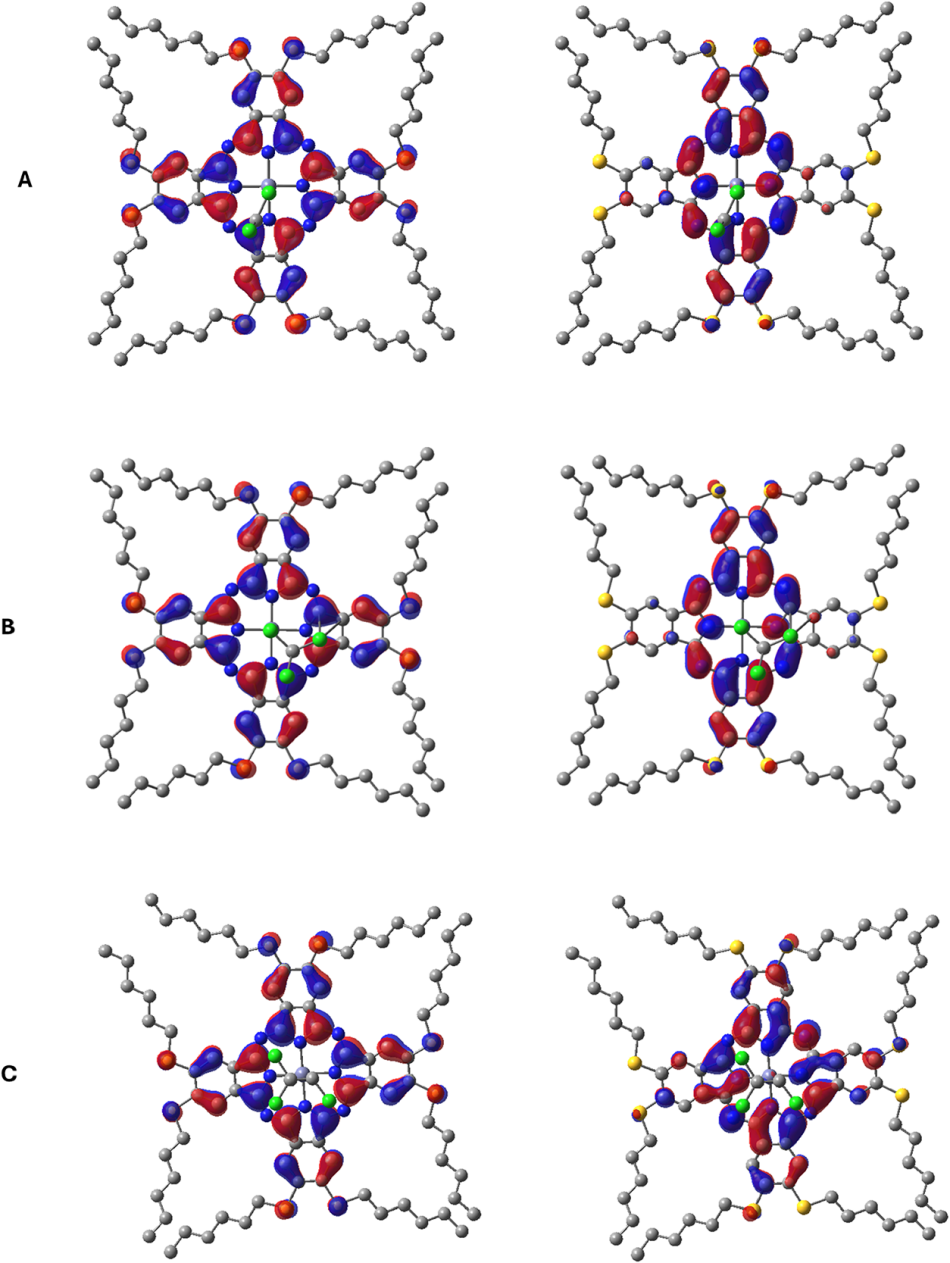
Frontier molecular orbitals of the most stable ZnPc2-B4–VOC complexes. For each complex, the left panel shows the HOMO and the right panel indicates the LUMO (A) dichloromethane, (B) chloroform, and (C) trichloroethylene.

## 4 Conclusions

The LC **ZnPc2-B4** molecule was prepared as a thin film sensor by using the spin coating method. The SPR technique was employed to collect the data during the interaction between the **ZnPc2-B4** thin film and a vapor molecule. Surface morphology of the **ZnPc2-B4** thin film was studied by AFM measurement. The change in optical and sensor parameters of the **ZnPc2-B4** thin film before and after the heating process was analyzed. DFT theory was applied to identify their interaction patterns.

AFM results showed that the **ZnPc2-B4** thin film yielded a compact and uniform film surface with a surface roughness value of 2.77 nm. Using the SPR results, the thickness values of the **ZnPc2-B4** thin film were 49.5 nm before heating and 43.5 nm after heating. The heating process decreased the film thickness. Refractive index values were calculated almost the same. Time-dependent SPR sensor measurements showed that **ZnPc2-B4** thin film responses were ordered as dichloromethane, chloroform, and trichloroethylene vapors. Dichloromethane vapor has the highest sensitivity before heating (0.2864x10^−3^% response/ppm), after heating (0.1015x10^−3^% response/ppm), and the lowest LOD before heating (11.53 ppm), after heating (32.51 ppm), and LOQ before heating (34.96 ppm), after heating (98.52 ppm) values than other vapors. Using DFT theory, optimized geometries of the most stable **ZnPc2-B4**–VOC complexes were calculated with binding free energies of −1.7 kcal/mol for dichloromethane, −3.8 kcal/mol for chloroform, and −5.7 kcal/mol for trichloroethylene vapors, respectively. This study can be concluded that thermal annealing modulates sensor performance, potentially enhancing stability at the expense of sensitivity. Zn···Cl, C···Cl, N···H, and Zn, with the nucleophilic carbon atom of vapor interactions playing an important role in the sensor response between the **ZnPc2-B4** thin film and chlorinated hydrocarbon vapors. Future work will be focused on the humidity effect at different RH values of the ZnPc2-B4 spun thin films due to the polar nature of some interactions.
